# Clinical and CT Features of HIV-Negative and HIV-Positive Patients with Abdominal Tuberculous Lymphadenopathy

**DOI:** 10.3390/diagnostics15222931

**Published:** 2025-11-20

**Authors:** Xiao-Ling Zhu, Sheng-Xiu Lv, Li Wen, Ran Li, Xue-Yan Liu, Guang-Xian Wang

**Affiliations:** 1 Department of Radiology, Banan Hospital, Chongqing Medical University, Chongqing 401320, China; 134072@hospital.cqmu.edu.cn (X.-L.Z.); lizeqian@hospital.cqmu.edu.cn (R.L.); 2 Department of Radiology, Chongqing Public Health Medical Center, Chongqing 400036, China; r202206@126.com; 3 Department of Radiology, Xinqiao Hospital, Third Military Medical University, Chongqing 400037, China; cqzdwl@tmmu.edu.cn

**Keywords:** CT, HIV, abdominal tuberculosis, extrapulmonary tuberculosis, lymphadenopathy

## Abstract

**Background**: The diagnosis of abdominal tuberculous lymphadenopathy (ATBL) remains challenging in clinical practice. Patients with ATBL and HIV infection may have atypical clinical and computed tomography (CT) features. The aim of this study was to investigate the impact of HIV infection on the clinical and CT features of ATBL patients. **Methods**: From January 2012 to March 2023, 178 patients with untreated ATBL were retrospectively analyzed. Patients with ATBL were classified into HIV-negative group (*n* = 152) and HIV-positive group (*n* = 26). In addition to the clinical characteristics of the patients, the features of ATBL (e.g., size and location) were evaluated via CT. The Mann–Whitney U test (for continuous variables) and Fisher’s exact test (for categorical variables) were used to compare clinical data and CT imaging features between the two groups. Missing values were handled using multiple imputation, and the Benjamini–Hochberg procedure was applied to control the false discovery rate (FDR) in multiple comparisons. Post hoc power analysis for key variables was performed. **Results**: Compared with the HIV-negative group, the HIV-positive group had older age, lower CD4^+^ T-cell counts, and larger ATBL diameter. The HIV-positive group also showed a stronger tendency for disease dissemination, with significantly higher rates of smear positivity, miliary pulmonary tuberculosis (PTB), and disseminated tuberculosis (TB). On CT imaging, the HIV-positive group had a higher frequency of ATBL involvement in the upper para-aortic region, portacaval space, and hepatogastric ligament. In contrast, abdominal distension was more common in the HIV-negative group. post hoc power analysis confirmed that most key variables had adequate statistical power (≥0.8), except for age (power = 0.597) and ATBL diameter (Power = 0.769). **Conclusions**: The clinical and CT features of ATBL differ significantly between HIV-negative and HIV-positive patients.

## 1. Introduction

TB remains a major, worldwide public health problem that is related to malnutrition, migration, economic and political pressures, drug abuse, and HIV positivity, among other variables. According to the WHO Global Tuberculosis Report 2022, the number of newly diagnosed TB cases declined in 2020 and 2021, likely due to disruptions from the COVID-19 pandemic. This decline implies a potential increase in undiagnosed and untreated cases in communities, further fueling the transmission of TB [1]. Notably, HIV-positive individuals are at a significantly higher risk of TB infection [2], and their clinical and radiographic manifestations of TB are often atypical, leading to delayed diagnosis and increased mortality [3]. The complex interaction between TB and HIV intensifies the global burden of both diseases.

TB can involve almost any organ, with the lungs as the most common site of infection. The abdomen is one of the primary locations for extrapulmonary TB (EPTB) [4,5], and abdominal TB (ATB) may affect the gastrointestinal tract, hepatobiliary system, peritoneum, and lymph nodes. ATBL is a common manifestation of abdominal tuberculosis (ATB); in up to 55% of patients with ATB, ATBL presents as an isolated manifestation without other organ involvement [6]. Timely diagnosis of ATBL is crucial, as untreated or delayed treatment is associated with high morbidity and mortality [3]. However, the diagnosis of ATB is often delayed because the clinical symptoms vary and are sometimes absent. This diagnostic dilemma is further compounded in HIV-positive patients, who frequently present with atypical clinical and imaging features, making early identification more difficult.

Imaging plays a pivotal role in the evaluation of ATBL. Although histopathological examination remains the gold standard for diagnosing TB, its application is often not feasible in clinical practice, particularly in cases of ATB. In this context, ultrasound is limited by bowel gas interference, while CT is the preferred modality for assessing the extent and characteristics of abdominal lymphadenopathy, despite its nonspecific findings [7]. ATBL usually presents as necrotic nodes in the mesenteric, peripancreatic, periportal, and upper para-aortic regions [6,8,9,10], and peripheral rim enhancement is the most common pattern [11]. Notably, HIV-positive patients often exhibit atypical CT findings of ATBL, which may obscure the diagnosis and delay treatment.

Recognition of these differences in CT between HIV-positive and HIV-negative patients may aid in earlier diagnosis and treatment. Hence, in this study, we aimed to compare the clinical and CT characteristics of HIV-positive and HIV-negative patients with ATBL.

## 2. Materials and Methods

### 2.1. Patients

This retrospective study was approved by the institutional ethics committee of each participating center (Banan Hospital, NO. BNLL-KY-2023-036; Chongqing Public Health Medical Center, NO. GWZX-2022-122603 and Xinqiao Hospital, NO. 2023011401). Informed consent was waived due to the retrospective nature of the study, and all patient data were de-identified.

Between January 2012 and March 2023, clinical and CT imaging records of 178 ATBL patients across three medical centers were retrospectively analyzed. To minimize patient selection bias across centers, the following standardized inclusion and exclusion criteria were applied uniformly. Inclusion criteria: Patients aged ≥18 years with a confirmed diagnosis of ATBL, no prior anti-TB treatment, and available abdominal contrast-enhanced CT images. Exclusion criteria: Patients aged <18 years, those who had received anti-TB therapy before enrollment, and those with incomplete imaging data that prevented comprehensive analysis.

Among the 178 patients included, ATBL was confirmed in 96 patients by either histological examination or percutaneous aspiration biopsy, in 41 patients by the growth of *Mycobacterium tuberculosis* (*M. tuberculosis*) on cultures of tissue or ascitic fluid and in 41 patients by a satisfactory therapeutic response to anti-TB treatment.

Hematogenous disseminated TB was considered when TB was found in at least 2 different organs or when a patient had active miliary PTB [6,8,12]. The patients were classified into two groups: the HIV-positive group and the HIV-negative group. HIV status was confirmed by Western blot analysis (TECAN, Switzerland). Three assessors collected patient clinical information (e.g., sex and age) from electronic medical records for further analysis.

### 2.2. Imaging Protocol and Analysis

All abdominal CT examinations were performed using 64-slice multidetector CT scanners (Philips Incisive, Philips Medical Systems, Best, The Netherlands; Revolution HD, GE Healthcare, Wauwatosa, WI, USA; LightSpeed VCT, GE Healthcare, Chicago, IL, USA).

All patients (except for 1 with intestinal obstruction and 10 with nausea and vomiting) fasted for 8 h and drank 800–1000 mL of water 30 min before the CT scan to distend the gastrointestinal tract. For patients with comorbid chronic kidney disease or chronic heart disease, a personalized water intake protocol was adopted (e.g., adjusting water volume, drinking in divided doses, or extending the interval between water intake). All patients underwent nonenhanced and enhanced scanning of the entire abdomen. A total of 80–100 mL of nonionic contrast medium (Visipaque 320, GE Healthcare; or Ioversol 350, Hengrui Pharmaceuticals, Lianyungang, China) was injected into the antecubital vein at a rate of 3–3.5 mL/s. After contrast medium was injected, the arterial phase, portal venous phase and delayed phase data were obtained at 25–30 s, 50–60 s and 120 s, respectively. All of the CT data were subsequently thinly sliced at a thickness of 0.625 mm or 0.5 mm.

The CT images were independently analyzed by two radiologists (one with 8 years of experience in abdominal imaging and the other with 15 years of experience in radiology diagnostics), and continuous data were recorded as average values for subsequent analyses. Any discrepancies in categorical data were re-evaluated by a third reader (who had 25 years of experience in radiology). The CT characteristics of lymphadenopathy, including size, anatomic location, enhancement pattern (peripheral rim, homogeneous, nonhomogeneous, homogeneous nonenhancement or mixed enhancement), and degree of enhancement (low, iso-, or high), were recorded. Lymph nodes were considered abnormal and included for measurement if the short-axis diameter was ≥10 mm or if they exhibited features such as peripheral rim enhancement, nonhomogeneous enhancement, or fusion. The largest short-axis diameter was measured for each node. In cases of confluent lesions, the observers attempted to delineate individual node boundaries for measurement; if this was not feasible, the maximum short-axis diameter of the fused mass on the axial image was recorded [13]. Lymph nodes are grouped into the following anatomic regions: the hepatogastric ligament, hepatoduodenal ligament, portacaval space, gastrosplenic ligament, greater omentum, mesentery, peripancreatic region, upper and lower para-aortic regions (demarcated by the upper edge of L3), iliac vessel regions, pelvic cavity and inguinal region [6,9]. Peripheral enhancement was defined as lymph nodes presenting with thick, irregular, or thin rims on contrast-enhanced CT images. Homogeneous enhancement was defined as necrotic and non-necrotic lymph nodes. Nonhomogeneous enhancement was considered when peripheral or homogeneous enhancement was not observed. Enhancement was considered mixed when the lymph nodes had at least two enhancement patterns. Skeletal muscle was used to evaluate the degree of enhancement of the lymph nodes; the evaluation method can be found in our previous article [14]. Notably, calcified lymph nodes were excluded. Moreover, the presence of concomitant lesions, including other organ lesions, ascites or peritonitis, was also recorded.

### 2.3. Statistical Analysis

The normality of continuous variables was assessed using the Shapiro–Wilk test. As the data were non-normally distributed, they were presented as median with interquartile range (IQR) and were compared using the Mann–Whitney U test. Categorical variables were expressed as frequency (%) and were compared using Fisher’s exact test. To minimize potential bias introduced by missing laboratory data, missing values were handled using multiple imputations.

To quantify the strength of associations, crude odds ratios (ORs) with 95% confidence intervals (CIs) for categorical variables and the Hodges-Lehmann median difference (Δ*M*) with 95% CIs for continuous variables were calculated. All ORs were computed with the HIV-negative group as the reference category (OR = 1.0). To control for Type I error inflation due to multiple comparisons, the FDR was controlled using the Benjamini–Hochberg procedure. Variables that remained significant after FDR correction were indicated with a dagger symbol (†), and an adjusted *p* value < 0.05 was considered statistically significant.

Statistical analysis was primarily performed using SPSS 25.0 (SPSS Inc., Chicago, IL, USA). Furthermore, owing to the sample size imbalance, a post hoc power analysis for key variables was conducted using R software (version 4.5.0; R Foundation for Statistical Computing, Vienna, Austria).

## 3. Results

### 3.1. Demographic Features and Clinical Features

Two hundred forty-four patients were diagnosed with ATBL, and 66 patients younger than 18 years of age or previously treated were excluded. The clinical and laboratory characteristics of the 178 patients (152 HIV-negative patients and 26 HIV-positive patients) are listed in Table 1. The median age for all patients was 29.5 years (interquartile range [IQR], 23.0–46.3 years). Males constituted 110 patients (61.8%). Only 7 patients (3.9%) were asymptomatic, while the majority (96.1%) presented with at least one clinical symptom. The clinical symptoms are displayed in Supplemental Table S1. The most frequent symptom was abdominal pain (60.1%), followed by abdominal distension (36.5%), fever (30.3%), and cough with sputum production (20.8%).

The HIV-positive group was significantly older than the HIV-negative group (Δ*M* = 8.0 years, 95% CI: 3.0, 15.0; adjusted *p* = 0.030). Abdominal distension remained significantly more common in the HIV-negative group (OR = 0.189, 95% CI: 0.054, 0.658, adjusted *p* = 0.036). However, the differences in sex distribution and abdominal pain between the groups were no longer statistically significant after correction (adjusted *p* = 0.190 and *p* = 0.085, respectively). Although fever was more prevalent in the HIV-positive group (46.2% vs. 27.6%), this difference was not significant (adjusted *p* = 0.244). No significant differences were observed for other symptoms such as cough with sputum production, weakness, or diarrhea (all adjusted *p* > 0.05).

Regarding the clinical history of the patients, the proportion of diabetic patients was higher in the HIV-positive group in the unadjusted analysis (raw *p* = 0.041), but this difference was no longer statistically significant after Benjamini–Hochberg correction (adjusted *p* = 0.17). Additionally, there were no statistically significant differences in the prevalence of other chronic comorbidities examined between the two groups (including malignant tumors, history of solid organ or hematopoietic stem cell transplantation, chronic liver disease, heart disease, pulmonary disease, and kidney disease), with all adjusted *p* > 0.05.

In summary, this study enrolled 178 ATBL patients, predominantly young adult males, with the vast majority presenting clinical symptoms. Comparative analysis revealed that HIV-positive patients were significantly older, while abdominal distension was significantly more common in HIV-negative patients. No statistically significant differences were observed in the remaining clinical symptom profiles or the prevalence of other chronic comorbidities between the two groups.

### 3.2. Patient Laboratory Features

Some laboratory data were missing: CD4^+^ T-cell counts were missing in 55 patients (30.9%), lymphocyte counts in 6 (3.4%), tuberculin skin test results in 6 (3.4%), and smear microscopy for acid-fast bacilli (AFB) in 9 (5.1%). Multiple imputation was used to handle all missing data, and the following analyses were all based on the imputed dataset.

The median CD4^+^ T-cell count was significantly lower in the HIV-positive group (Δ*M* = −214 cells/µL, 95% CI: −260, −165, adjusted *p* = 0.015). Similarly, the proportion of AFB smear-positive patients was significantly higher in the HIV-positive group compared to the HIV-negative group (OR = 5.167, 95% CI: 2.157, 12.373, adjusted *p* = 0.015).

Conversely, no statistically significant difference was observed in the tuberculin skin test results and lymphocyte counts between the HIV-positive and HIV-negative groups.

In summary, the laboratory characteristics of HIV-positive ATBL patients were marked by severe immunosuppression and a higher bacterial load, as evidenced by significantly lower CD4^+^ T-cell counts and a higher positive rate of AFB smear. In contrast, there were no significant differences in lymphocyte counts and tuberculin skin test reactivity between the two groups.

### 3.3. CT Characteristics of ATBL

CT characteristics of ATBL are detailed in Table 2 and Supplemental Table S2. Among all cases, the mesentery was the most frequently involved site (87.6%), followed by the upper para-aortic region (55.6%) and lower para-aortic region (33.1%). Homogeneous enhancement was the most common enhancement pattern (47.8%), followed by mixed enhancement (37.6%). Regarding co-involvement of other sites, pulmonary involvement was the most prevalent (68.5%), followed by gastrointestinal involvement (50.0%) and pleural involvement (23.0%). Serous cavity involvement was also relatively common, with ascites (58.4%), pleural effusion (35.4%), and greater omental infiltration (45.5%). Only 7 patients (3.9%) presented with isolated ATBL without other organ involvement, while 2 patients (1.1%) had single-site ATBL.

**Figure 1:**
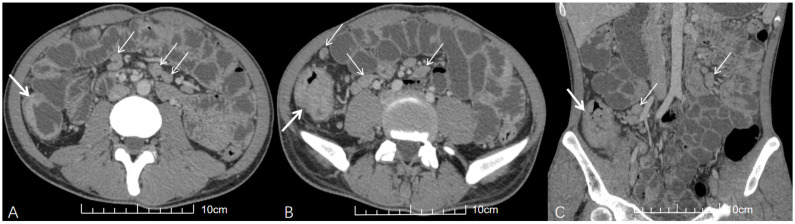
A young HIV-negative patient with abdominal pain and diarrhea for 1 month. (**A**,**B**) Axial and (**C**) coronal contrast-enhanced abdominal CT (venous phase) images demonstrate multiple enlarged lymph nodes in the mesentery with homogeneous enhancement and no fusion (thin white arrows). Concurrent ileocecal intestinal TB is evidenced by circumferential wall thickening (thick white arrows in (**A**–**C**)).

**Figure 2:**
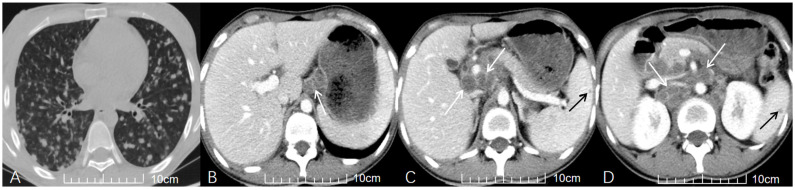
A young HIV-positive patient with recurrent fever for one month. (**A**) Axial non-contrast chest CT reveals subacute hematogenous disseminated PTB, manifesting as diffuse nodules (2–5 mm) in both lungs. (**B**–**D**) Axial contrast-enhanced abdominal CT (venous phase) demonstrates multiple enlarged lymph nodes in the hepatoduodenal ligament, portacaval space, and upper para-aortic region, exhibiting peripheral rim enhancement and fusion (white arrows in (**B**), (**C**), (**D**), respectively). Concurrent splenic TB is indicated by the presence of low-attenuation nodules (black arrows in (**C**,**D**)).

Distribution analysis revealed that ATBL in the HIV-negative group most often involved the mesentery (Figure 1), followed by para-aortic regions (upper and lower) and the hepatoduodenal ligament. In contrast, the HIV-positive group showed predominant involvement of the upper para-aortic region, mesentery, and portacaval space, followed by the hepatoduodenal ligament, hepatogastric ligament, and lower para-aortic region. Notably, the HIV-positive group demonstrated significantly higher involvement rates in the upper para-aortic region (OR = 12.320, 95% CI: 2.813, 53.966, adjusted *p* = 0.015), portacaval space (OR = 7.648, 95% CI: 3.123, 18.729, adjusted *p* = 0.015), and hepatogastric ligament (OR = 4.067, 95% CI: 1.710, 9.671, adjusted *p* = 0.025) compared to the HIV-negative group (Figure 2).

Regarding morphological characteristics, the HIV-positive group had significantly larger median ATBL diameter (2.25 cm vs. 1.60 cm), with statistical significance (Δ*M* = 0.50 cm, 95% CI: 0.20, 0.90, adjusted *p* = 0.030). Lymph node fusion into masses was also more commonly observed in the HIV-positive group (65.4% vs. 36.2%; raw *p* = 0.008), though this difference did not maintain statistical significance after multiple comparison correction (adjusted *p* = 0.056). As for enhancement characteristics, homogeneous enhancement was the most common enhancement pattern in the HIV-negative group (Figure 1), followed by mixed and peripheral rim enhancement. However, mixed enhancement was the most common enhancement pattern in the HIV-positive group, followed by homogeneous enhancement and peripheral rim enhancement, but the HIV-positive group showed a higher proportion of peripheral rim enhancement (Figure 2). No significant differences in enhancement patterns or degree were observed between the two groups after multiple corrections.

Notably, the HIV-positive group had a significantly higher incidence of disseminated TB (OR = 4.653, 95% CI: 1.841, 11.759, adjusted *p* = 0.015) and miliary PTB (OR = 9.165, 95% CI: 3.516, 23.886, adjusted *p* = 0.015) compared with the HIV-negative group. Additionally, pericardial TB was only detected in the HIV-positive group (7.7%) and absent in the HIV-negative group, but this difference became non-significant after adjustment.

In summary, ATBL most commonly involves the mesentery and para-aortic regions, with homogeneous or mixed enhancement, and is frequently complicated by pulmonary and gastrointestinal TB. Compared with the HIV-negative group, the HIV-positive group is more prone to lymph node involvement in the upper para-aortic region, portacaval space, and hepatogastric ligament, with larger diameters and higher incidences of disseminated and miliary PTB.

### 3.4. Post Hoc Power Analysis

Given the small sample size of the HIV-positive group (*n* = 26), a post hoc power analysis was performed for key variables with statistically significant differences (adjusted *p* < 0.05) in the univariate analysis. As shown in Table 3, except for age (power = 0.597) and ATBL diameter (power = 0.769), the power of all other key variables (CD4^+^ T-cell count, miliary PTB, upper para-aortic region involvement, portacaval space involvement, hepatogastric ligament involvement, abdominal distension, AFB smear positivity, and disseminated TB) all exhibited sufficient statistical power (≥0.8). This indicates that the study had sufficient statistical power for most key variables.

### 3.5. Visualization of Core Clinical-Radiologic Associations

**Figure 3:**
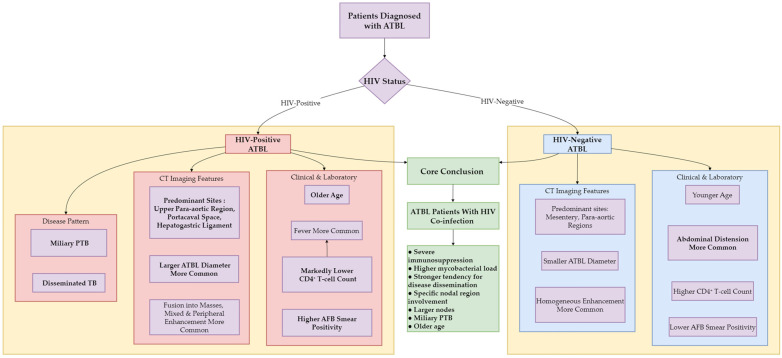
Clinical-radiologic differences in ATBL patients by HIV status.

To intuitively integrate the key findings of clinical symptoms, laboratory indicators, CT features, and statistical analyses, we constructed a summary diagram (Figure 3) that illustrates the associations between HIV status and ATBL characteristics. This diagram provides a concise overview of the distinct profiles of the two groups, facilitating a comprehensive understanding of the study results.

## 4. Discussion

### 4.1. Demographic Features and Clinical Symptoms

Regarding demographic features, our study showed that older age was associated with HIV infection. In the HIV-negative group, 57.9% (88/152) of patients were younger (≤30 years), in contrast to the HIV-positive group in which 73.1% of the patients were over 30 years old. This finding is consistent with previous study results indicating that EPTB particularly occurs in younger patients [12,15,16]. The older age observed in the HIV-positive group may reflect local epidemiological patterns of HIV infection. It is important to note, however, that the post hoc power for age was suboptimal (0.597), indicating a non-negligible risk of a Type II error and suggesting this finding requires cautious interpretation and validation in larger studies. In terms of gender distribution, although previous studies have suggested that endocrine factors may pose a higher risk of EPTB in females than in males [17], this study had a male majority (61.87%), similar to previous studies [15,16]. This male predominance was more pronounced in the HIV-positive group. Although gender showed no statistically significant difference between the two groups after correction, this trend suggests that gender might be a potential associated factor for ATBL and HIV co-infection. Further prospective studies with larger sample sizes are needed to verify its independence.

At the clinical symptom level, the clinical manifestations of ATB can be asymptomatic or cause nonspecific symptoms [10,18]. In this study, abdominal pain and abdominal distension were the most common initial symptoms. Similarly, Kapoor et al. [19] analyzed features of ATB and reported that abdominal pain was the most common symptom, followed by abdominal distension, which is consistent with our findings. However, it is noteworthy that in the HIV-positive group, the most common initial symptom of ATBL was fever, followed by abdominal pain; in contrast, abdominal distension was significantly more prevalent in the HIV-negative group. This suggests that HIV co-infection may shift the ATBL symptom profile toward systemic toxicity (e.g., fever) and away from local abdominal signs such as distension. This phenomenon may be related to HIV-associated immunosuppression facilitating hematogenous dissemination of *M. tuberculosis*, triggering a systemic inflammatory response. While weakening the host’s granulomatous response, it also reduces the degree of focal inflammation in the intestinal wall and surrounding areas, thereby diminishing localized gastrointestinal irritative symptoms like abdominal distension. This shift highlights the atypical clinical features of ATBL in immunocompromised individuals, necessitating clinicians’ attention to this symptomatic difference during diagnosis to avoid missing ATBL in HIV-positive patients due to the “absence of abdominal distension.”

In addition, before multiple corrections, the prevalence of diabetes mellitus was higher in the HIV-positive group than in the HIV-negative group; however, the difference was not statistically significant after adjustment (adjusted *p* = 0.17). Nevertheless, this finding still suggests a potential association between HIV and diabetes. This association may be related to the use of antiretroviral drugs in HIV treatment, which can cause weight gain and obesity, thereby increasing the risk of diabetes [20]. Diabetes itself can further impair immune function and exacerbate the severity of TB infection. The two conditions may act as mutual adverse factors, and the association between HIV and diabetes mellitus requires further validation in prospective cohort studies.

### 4.2. Laboratory Features

In terms of laboratory parameters, the CD4^+^ T-cell count in the HIV-positive group was significantly lower than that in the HIV-negative group, indicating severe immunosuppression in HIV-positive patients. Additionally, the positive rate of AFB smear was significantly higher in the HIV-positive group, suggesting a greater tendency for dissemination in ATBL patients with HIV co-infection. The incidence of miliary PTB was also significantly higher in the HIV-positive group than in the HIV-negative group. These two indicators collectively reflect the impairment of immune function and increased *M. tuberculosis* load caused by HIV infection.

### 4.3. CT Imaging Analysis

The mesentery, upper para-aortic region, periportal area, and peripancreatic region are typical sites of ATBL involvement [10,18,21], reflecting the lymphatic drainage pattern of the small intestine. In this study, we found ATBL to be more common in the mesentery, upper and lower para-aortic regions, hepatoduodenal ligament, hepatogastric ligament, and portacaval space. Specifically, the involvement rates of the upper para-aortic region, portacaval space, and hepatogastric ligament were significantly higher in the HIV-positive group compared to the HIV-negative group, whereas the HIV-negative group predominantly involved the mesentery and upper para-aortic region. A potential explanation lies in the altered dissemination pattern associated with HIV. HIV-negative patients with normal immune function typically have *M. tuberculosis* confined to the intestines and local lymphatic system, hence mesenteric lymph nodes are usually involved. In contrast, HIV-positive patients, due to immunosuppression, experience *M. tuberculosis* breaching local barriers and disseminating hematogenously to systemic lymphatic hub areas. This aligns with and mutually confirms the significantly higher incidence of miliary PTB and disseminated TB observed in the HIV-positive group in our study.

In terms of lymph node morphology, the diameter of ATBL was significantly larger in the HIV-positive group than in the HIV-negative group, consistent with the pathological process of continuous *M. tuberculosis* proliferation and uncontrolled lymph node growth in an immunosuppressed state. However, the post hoc power for ATBL diameter was 0.769, this suggests that the stability and generalizability of this particular finding might be limited by the sample size. Although the proportion of lymph nodes fused into masses was higher in the HIV-positive group compared to the negative group, the difference was not statistically significant after multiple comparison correction. This lack of significance is presumably due to insufficient statistical power caused by the small sample size of the HIV-positive group (*n* = 26), and verification with larger sample sizes is needed.

The CT characteristics of ATBL typically include peripheral enhancement with central low attenuation owing to perinodal granulation tissue and central caseous necrosis [6,10,18]; although this is not pathognomonic, it is a useful sign and can be readily observed on CT [22]. Interestingly, although scholars in previous studies [6,9] reported that peripheral enhancement was the most common characteristic of ATBL, this study revealed that only 17 patients had ATBL with simple peripheral enhancement, and it was more prone to appear in the HIV-positive group. In contrast, homogeneous enhancement was the most common enhancement pattern in this study and was more common in the HIV-negative group than in the HIV-positive group; this finding added to the diagnostic difficulties and differed from the poor nodal enhancement that is usually observed in HIV-positive patients [23]. The high proportion of homogeneous enhancement in this study may be related to the fact that most HIV-negative patients had normal immune function and milder focal inflammatory responses. Further studies are required to confirm this observation.

### 4.4. Co-Involvement of Other Sites

In this study, 171 (96.1%) ATBL patients had involvement in other sites, mainly the lungs and gastrointestinal tract, while only 7 (3.9%) had isolated ATBL. This differs from previous reports that 55% of ATBL cases present as isolated lesions [8], which may be attributed to the inclusion of mostly symptomatic patients with a higher proportion of disseminated lesions in our study. Pericardial TB is a rare form of TB (observed in only 2 HIV-positive patients), suggesting it may be a unique manifestation of HIV co-infection. However, due to the limited sample size, further studies are needed to explore this association. Disseminated TB is known to be more common in HIV-positive patients than in HIV-negative patients [12], which is consistent with our results. In this study, miliary PTB was more prevalent in the HIV-positive group and was more likely to involve abdominal organs through hematogenous spread [8]. Both miliary PTB and disseminated TB were diagnosed significantly more often in the HIV-positive group.

## 5. Limitations

This study has several limitations. First, this was a retrospective study with a small sample size, especially among HIV-positive patients, which may have affected our analysis. A multicenter study with a large sample size is necessary in the future. Second, we did not perform multivariable logistic regression due to the limited sample size of the HIV-positive group (*n* = 26) and the risk of overfitting, which precluded identification of independent predictors. Third, the different CD4^+^ T-cell counts of HIV-positive patients count may affect the clinical and CT characteristics of ATBL, so more in-depth stratified analysis is needed. Fourth, although multiple imputations were applied to handle missing laboratory data, the incompleteness of these data remains a potential source of statistical bias. Fifth, this study focused only on patients with ATBL and was not applicable to other sites of TB. Sixth, the time between symptom onset and CT examination varied among patients, possibly resulting in different ATBL characteristics on CT.

## 6. Conclusions

In areas with high TB prevalence, patients presenting with abdominal pain and distension with or without fever, and CT scans showing multiple regional abdominal lymph node enlargements with marginal or uniform enhancement, may suggest the possibility of ATBL.

HIV-positive patients more frequently presented with disseminated disease, characterized by involvement of specific abdominal nodal regions (the upper para-aortic region, portacaval space, and hepatogastric ligament), larger lymph node size, and a higher prevalence of miliary PTB and AFB smear positivity. These features, combined with severe immunosuppression indicated by significantly lower CD4^+^ T-cell counts, suggest a more extensive disease burden. It should be noted that the associations for older age and larger ATBL diameter, while statistically significant in our cohort, had limited statistical power in post hoc analysis and warrant confirmation in larger prospective studies.

In summary, the clinical and CT features of ATBL differ significantly based on patients’ HIV status. Recognizing this distinct clinical and imaging profile for ATBL in HIV-positive patients may heighten clinical suspicion. This awareness may heighten clinical suspicion for HIV co-infection and disseminated TB, potentially facilitating timely diagnostic work-up for this vulnerable population.

## Figures and Tables

**Table 1:** Clinical and laboratory features of patients with ATBL by difference in HIV group.

	HIV-Negative Group (*n* = 152)	HIV-Positive Group (*n* = 26)	ORs/Δ*M* [95% CIs]	Raw *p*	Adjusted *p*
^†^ Age (years)	27.0 (22.0–46.0)	42.5 (29.8–50.3)	8.0 [3.0, 15.0]	0.003	0.030
Males	89 (58.6%)	21 (80.8%)	2.973 [1.064, 8.305]	0.048	0.203
Smoking history	48 (31.6%)	10 (38.5%)	1.354 [0.572, 3.203]	0.503	1.000
Alcohol intake	31 (20.4%)	4 (15.4%)	0.710 [0.228, 2.210]	0.790	1.000
Clinical Symptoms					
Abdominal pain	97 (63.8%)	10 (38.5%)	0.354 [0.150, 0.835]	0.015	0.095
^†^ Abdominal distension	62 (40.8%)	3 (11.5%)	0.189 [0.054, 0.658]	0.004	0.036
Diabetes mellitus	3 (2.0%)	3 (11.5%)	6.478 [1.232, 34.052]	0.041	0.182
Malignant tumor	4 (2.6%)	0 (0%)	N/A ^	1.000	1.000
Solid-organ or hematopoietic stem cell transplantation	1 (0.7%)	0 (0%)	N/A ^	1.000	1.000
Chronic liver disease	22 (14.5%)	6 (23.1%)	1.773 [0.640, 4.907]	0.255	0.672
Chronic heart disease	4 (2.6%)	0 (0%)	N/A ^	1.000	1.000
Chronic pulmonary disease	2 (1.3%)	0 (0%)	N/A ^	1.000	1.000
Chronic kidney disease	5 (3.3%)	0 (0%)	N/A ^	1.000	1.000
^†^ CD4^+^ T-cell counts (cells/µL) *	301 (207–383)	66 (22–130)	−214 [−260, −165]	<0.001	0.015
Lymphocyte counts (×10^9^/L) ^#^	0.877 (0.623–1.253)	0.860 (0.415–1.490)	0.067 [−0.226, 0.308]	0.599	1.000
Tuberculin skin test (positive) ^&^	93 (61.2%)	10 (38.5%)	0.397 [0.169, 0.932]	0.034	0.160
^†^ Smear microscopy test for AFB (positive) ^$^	28 (18.4%)	14 (53.8%)	5.167 [2.157, 12.373]	<0.001	0.015

ATBL, abdominal tuberculous lymphadenopathy; AFB, acid-fast bacilli; ^†^, Significant after Benjamini–Hochberg correction for multiple comparisons (adjusted *p* < 0.05); ^, OR not applicable due to a zero cell count; *, 55 patients had missing data; ^#^, 6 patients had missing data; ^&^, 6 patients had missing data; ^$^, 9 patients had missing data.

**Table 2:** CT findings of patients with ATBL by difference in HIV group.

	HIV-Negative Group (*n* = 152)	HIV-Positive Group *(n* = 26)	ORs/Δ*M* [95% CIs]	Raw *p*	Adjusted *p*
Pericardial TB	0 (0%)	2 (7.7%)	N/A ^	0.021	0.109
^†^ Miliary PTB	13 (8.6%)	12 (46.2%)	9.165 [3.516, 23.886]	<0.001	0.015
Active PTB	94 (61.8%)	19 (73.1%)	1.675 [0.663, 4.229]	0.378	0.812
Pleural effusion	54 (35.5%)	12 (46.2%)	1.556 [0.672, 3.602]	0.380	0.812
Ascites	90 (59.2%)	14 (53.8%)	0.804 [0.348, 1.855]	0.669	1.000
Peritonitis	85 (55.9%)	16 (61.5%)	1.261 [0.538, 2.958]	0.671	1.000
Greater omentum infiltration	75 (49.3%)	6 (23.1%)	0.308 [0.117, 0.809]	0.018	0.107
^†^ Disseminated TB	56 (36.8%)	19 (73.1%)	4.653 [1.841, 11.759]	0.001	0.015
Hepatosplenomegaly	8 (5.3%)	2 (7.7%)	1.500 [0.300, 7.494]	0.642	1.000
Location					
^†^ Upper para-aortic regions	75 (49.3%)	24 (92.3%)	12.320 [2.813, 53.966]	<0.001	0.015
Lower para-aortic regions	48 (31.6%)	11 (42.3%)	1.589 [0.679, 3.717]	0.367	0.812
Hepatoduodenal ligament	35 (23.0%)	13 (50.0%)	3.343 [1.420, 7.872]	0.007	0.057
^†^ Portacaval space	23 (15.1%)	15 (57.7%)	7.648 [3.123, 18.729]	0.001	0.015
Peripancreatic region	24 (15.8%)	4 (15.4%)	0.970 [0.307, 3.066]	1.000	1.000
Mesentery	135 (88.8%)	21 (80.8%)	0.529 [0.176, 1.586]	0.328	0.775
Iliac vessels regions	18 (11.8%)	3 (11.5%)	0.971 [0.265, 3.562]	1.000	1.000
^†^ Hepatogastric ligament	30 (19.7%)	13 (50.0%)	4.067 [1.710, 9.671]	0.002	0.025
Gastrosplenic ligament	3 (2.0%)	0 (0%)	N/A ^	1.000	1.000
Greater omentum	12 (7.9%)	1 (3.8%)	0.467 [0.058, 3.750]	0.695	1.000
Pelvic cavity	1 (0.7%)	0 (0%)	N/A ^	1.000	1.000
Inguinal region	2 (1.3%)	0 (0%)	N/A ^	1.000	1.000
Single ATBL	2 (1.3%)	0 (0%)	N/A ^	1.000	1.000
^†^ Diameter of ATBL (cm)	1.60 (1.20–2.10)	2.25 (1.60–2.85)	0.50 [0.20, 0.90]	0.003	0.030
Fused into mass	55 (36.2%)	17 (65.4%)	3.331 [1.391, 7.976]	0.008	0.059
Enhancement pattern					
Peripheral rim	11 (7.2%)	6 (23.1%)	3.845 [1.281, 11.546]	0.022	0.109
Homogeneous	79 (52.0%)	6 (23.1%)	0.277 [0.105, 0.729]	0.010	0.068
Nonhomogeneous	8 (5.3%)	0 (0%)	N/A ^	0.606	1.000
Homogeneous nonenhancement	1 (0.7%)	0 (0%)	N/A ^	1.000	1.000
Mixed enhancement	53 (34.9%)	14 (53.8%)	2.179 [0.941, 5.049]	0.080	0.284
Enhancement degree					
Low density	18 (11.8%)	7 (26.9%)	2.743 [1.013, 7.429]	0.062	0.251
Iso-density	49 (32.2%)	10 (38.5%)	1.314 [0.556, 3.105]	0.653	1.000
High density	101 (66.4%)	15 (57.7%)	0.689 [0.295, 1.607]	0.383	0.812

Abbreviations and symbols as defined in Table 1. TB, tuberculosis.

**Table 3:** Effect sizes and post hoc power of variables with significant differences.

Variable	Actual Power **
CD4^+^ T-cell counts	1.000
Upper para-aortic regions	0.998
Portacaval space	0.992
Miliary PTB	0.989
Smear microscopy test for AFB (positive)	0.948
Disseminated TB	0.940
Abdominal distension	0.904
Hepatogastric ligament	0.865
Diameter of ATBL	0.769
Age	0.597

** Adequate power: ≥0.8; Inadequate power: <0.8.

## Data Availability

The original contributions presented in the study are included in the article, further inquiries can be directed to the corresponding author.

## Supplementary Materials

The following supporting information can be downloaded at: https://www.mdpi.com/article/10.3390/diagnostics15222931/s1. Table S1. Comparison of clinical symptoms between HIV-positive and HIV-negative patients with ATBL. Table S2. Comparison of other tuberculous site involvement between HIV-positive and HIV-negative patients with ATBL.

## Abbreviations

ATBL	abdominal tuberculous lymphadenopathy
TB	tuberculosis
PTB	pulmonary TB
EPTB	extrapulmonary TB
ATB	abdominal TB
AFB	acid-fast bacilli
*M.* *tuberculosis*	*Mycobacterium tuberculosis*
