# Supplementary material for: Clinical and CT Features of HIV-Negative and HIV-Positive Patients with Abdominal Tuberculous Lymphadenopathy
Source: Diagnostics (Basel). 2025 Nov 20;15(22):2931. doi: 10.3390/diagnostics15222931 (PMC12651784; doi:10.3390/diagnostics15222931)
Supplement: Supplementary file 1 [file diagnostics-15-02931-s001.zip › diagnostics-3913424-supplementary.pdf]

diagnostics

MDPI

Supplemental Table 1. Comparison of clinical symptoms between HIV-positive and HIV-negative patients with ATBL

Clinical Symptoms	HIV-negative group (*n*=152)	HIV-positive group (*n*=26)	ORs [95% CIs]	Raw *P*	Adjusted *P*
Asymptomatic	6 (3.9%)	1 (3.8%)	0.973 [0.112, 8.432]	1.000	1.000
Abdominal pain	97 (63.8%)	10 (38.5%)	0.354 [0.150, 0.835]	0.015	0.095
†Abdominal distension	62 (40.8%)	3 (11.5%)	0.189 [0.054, 0.658]	0.004	0.036
Diarrhea	14 (9.2%)	5 (19.2%)	2.347 [0.766, 7.190]	0.162	0.465
Haematochezia	7 (4.6%)	0 (0%)	N/A^	1.000	1.000
Constipation	2 (1.3%)	0 (0%)	N/A^	1.000	1.000
Increased frequency of defecation	1 (0.7%)	0 (0%)	N/A^	1.000	1.000
Rectal tenesmus	2 (1.3%)	0 (0%)	N/A^	1.000	1.000
The changes of character of stool	5 (3.3%)	0 (0%)	N/A^	1.000	1.000
Intestinal obstruction	1 (0.7%)	0 (0%)	N/A^	1.000	1.000
Fever	42 (27.6%)	12 (46.2%)	2.245 [0.960, 5.247]	0.067	0.259
Loss of appetite	28 (18.4%)	6 (23.1%)	1.329 [0.489, 3.612]	0.592	1.000
Night sweat	24 (15.8%)	3 (11.5%)	0.696 [0.193, 2.501]	0.770	1.000
Weak	15 (9.9%)	5 (19.2%)	2.175 [0.716, 6.608]	0.179	0.498
Loss of weight	12 (7.9%)	1 (3.8%)	0.467 [0.058, 3.750]	0.695	1.000
Cough and sputum production	30 (19.7%)	7 (26.9%)	1.498 [0.577, 3.890]	0.435	0.900
Breathless	7 (4.6%)	1 (3.8%)	0.829 [0.098, 7.027]	1.000	1.000
Chest pain	4 (2.6%)	0 (0%)	N/A^	1.000	1.000
Tightness in the chest	1 (0.7%)	0 (0%)	N/A^	1.000	1.000
Nausea and vomiting	9 (5.9%)	1 (3.8%)	0.636 [0.07, 5.238]	1.000	1.000
Headache	1 (0.7%)	1 (3.8%)	6.040 [0.366, 99.717]	0.272	0.672
Dizzy	0 (0%)	1 (3.8%)	N/A^	0.146	0.448

Abbreviations and symbols as defined in Table 1.

Supplemental Table 2. Comparison of other tuberculous site involvement between HIV-positive and HIV-negative patients with ATBL

Other sites of TB	HIV-negative group (*n*=152)	HIV-positive group (*n*=26)	ORs [95% CIs]	Raw *P*	Adjusted *P*
No other sites of TB	5 (3.3%)	2 (7.7%)	2.450 [0.450, 13.353]	0.271	0.672
Pulmonary	103 (67.8%)	19 (73.1%)	1.291 [0.509, 3.276]	0.655	1.000
Pleura	32 (21.1%)	9 (34.6%)	1.985 [0.809, 4.869]	0.137	0.435
Mediastinum	2 (1.3%)	2 (7.7%)	6.250 [0.840, 46.495]	0.102	0.349
Pericardial	0 (0%)	2 (7.7%)	N/A^	0.021	0.109
Chest Wall	2 (1.3%)	0 (0%)	N/A^	1.000	1.000
Intracranial	6 (3.9%)	2 (7.7%)	2.028 [0.387, 10.638]	0.331	0.775
Neck	8 (5.3%)	4 (15.4%)	3.273 [0.909, 11.787]	0.078	0.285
Fossa axillaris	3 (2.0%)	2 (7.7%)	4.139 [0.657, 26.071]	0.155	0.460
Throat	1 (0.7%)	0 (0%)	N/A^	1.000	1.000
Pharynx	3 (2.0%)	0 (0%)	N/A^	1.000	1.000
Gastrointestinal tract	80 (52.6%)	9 (34.6%)	0.476 [0.200, 1.136]	0.136	0.435
Liver	4 (2.6%)	0 (0%)	N/A^	1.000	1.000
Spleen	16 (10.5%)	0 (0%)	N/A^	1.000	1.000
Reproductive system	7 (4.6%)	0 (0%)	N/A^	1.000	1.000
Urinary system	4 (2.6%)	1 (3.8%)	1.480 [0.159, 13.790]	0.550	1.000
Abdominal wall	5 (3.3%)	0 (0%)	N/A^	1.000	1.000

Psoas major	1 (0.7%)	1 (3.8%)	6.040 [0.366, 99.717]	0.272	0.672
Bone	6 (3.9%)	0 (0%)	N/A^	1.000	1.000
Skin	1 (0.7%)	0 (0%)	N/A^	1.000	1.000

Abbreviations and symbols as defined in Table 1. TB, tuberculosis. TB, tuberculosis.
